# Desmoglein2 Regulates Claudin2 Expression by Sequestering PI-3-Kinase in Intestinal Epithelial Cells

**DOI:** 10.3389/fimmu.2021.756321

**Published:** 2021-09-30

**Authors:** Natalie Burkard, Michael Meir, Felix Kannapin, Christoph Otto, Maximilian Petzke, Christoph-Thomas Germer, Jens Waschke, Nicolas Schlegel

**Affiliations:** ^1^ Department of General, Visceral, Transplant, Vascular and Pediatric Surgery University Hospital Würzburg, Würzburg, Germany; ^2^ Institute of Anatomy and Cell Biology, Department I, Ludwig-Maximilians-Universität München, Munich, Germany

**Keywords:** Claudin2, Dsg2, inflammation, intestinal barrier, PI-3-kinase, inflammatory bowel disease, desmosome, tight junction

## Abstract

Inflammation-induced reduction of intestinal desmosomal cadherin Desmoglein 2 (Dsg2) is linked to changes of tight junctions (TJ) leading to impaired intestinal epithelial barrier (IEB) function by undefined mechanisms. We characterized the interplay between loss of Dsg2 and upregulation of pore-forming TJ protein Claudin2. Intraperitoneal application of Dsg2-stablising Tandem peptide (TP) attenuated impaired IEB function, reduction of Dsg2 and increased Claudin2 in DSS-induced colitis in C57Bl/6 mice. TP blocked loss of Dsg2-mediated adhesion and upregulation of Claudin2 in Caco2 cells challenged with TNFα. In Dsg2-deficient Caco2 cells basal expression of Claudin2 was increased which was paralleled by reduced transepithelial electrical resistance and by augmented phosphorylation of AKT^Ser473^ under basal conditions. Inhibition of phosphoinositid-3-kinase proved that PI-3-kinase/AKT-signaling is critical to upregulate Claudin2. In immunostaining PI-3-kinase dissociated from Dsg2 under inflammatory conditions. Immunoprecipitations and proximity ligation assays confirmed a direct interaction of Dsg2 and PI-3-kinase which was abrogated following TNFα application. In summary, Dsg2 regulates Claudin2 expression by sequestering PI-3-kinase to the cell borders in intestinal epithelium.

## Introduction

The intestinal epithelium provides a selective barrier which separates the gut lumen containing nutrients, commensal bacteria and pathogens from the inner part of the body (1). Loss of intestinal epithelial barrier (IEB) integrity is known to contribute to the pathogenesis of inflammatory bowel diseases (2, 3).

Under basal condition the intestinal mucosa is lined by a single layer of polarized epithelial cells (enterocytes) that are sealed by tight junctions (TJ) and held together by adherens junctions and desmosomes (3–5). Adherens junctions are composed of E-cadherins which are tethered to the actin cytoskeleton while desmosomes in the intestine are composed of the desmocadherins desmoglein2 (Dsg2) and desmocollin2 (Dsc2) (3, 6). Both are linked to the intermediate filament system *via* various adapter proteins. Previously, it was reported that desmosomal integrity is not only essential to maintain the intestinal epithelial barrier under basal conditions but it is meanwhile established that loss of Dsg2 significantly contributes to inflammation-induced breakdown of the gut barrier in inflammatory bowel diseases (6–11). Desmosomes are known to stabilize IEB function by strengthening intercellular adhesion. In addition they are increasingly recognized as signaling hubs that mediate a variety of signals linked to cell proliferation, apoptosis and barrier regulation (3, 11–16).

Beside the growing evidence for the importance of desmosomal integrity to maintain the IEB in health and disease, it is well established that the main diffusion barrier within the junctional complex is formed by tight junctions that consist of various transmembrane proteins including claudins and occludin which are typically found at the most apical part of the membranes (5). The majority of the claudins that are typically present in the intestine such as claudin1, 3, 4, 5, 7, and 8 exert barrier-sealing properties. In contrast, Claudin2 which is strongly upregulated in intestinal inflammation is a pore-forming claudin which increases the permeability for cations like Na^+^, K^+^, Li^+^ and water (9, 10, 17–19). Upregulation of Claudin2 has been linked to diarrhea as typical clinical symptom of gut barrier changes. Under basal conditions Claudin2 is largely absent in the adult colon tissue whereas it has been described to be expressed in the human small intestine along the crypt-villus axis especially in the crypts (20). It is known that Claudin2 is increased following stimulation of enterocytes with cytokines such as TNFα, which is also regarded as a key cytokine contributing to mucosal injury in inflammatory bowel diseases (18, 21, 22).

Many studies meanwhile described a close correlation between the integrity of desmosomes and tight junctions (23–25). In line with this, we found a correlation between inflammation-induced loss of Dsg2 and increased Claudin2 expression in patients with Crohn’s disease and *in vitro* in a previous study. Interestingly, we observed that restoration of Dsg2-mediated adhesion using a Dsg-linking tandem peptide (TP) blocked TNFα-induced upregulation of Claudin2 (10). Based on this, we tested here whether Dsg2-mediated adhesion/signalling may be directly involved in the regulation of Claudin2.

## Materials and Methods

### Test Reagents

TNFα (Biomol, Hamburg, Germany) was used at 100 ng/ml (10). Tandem Peptide was used at 20 µM *in vitro* and 10 µM *in vivo* (Bachem, Bubendorf, Switzerland). The PI-3-kinase inhibitor LY294002 was used at 20 µM (Millipore, Darmstadt, Germany). Dsg2 binding was blocked by using a Dsg2 specific monoclonal mouse antibody directed against the third extracellular repeat domains of Dsg2 (anti-Dsg2^EC^) (clone 10G11, sodium azide free, Progen, Heidelberg, Germany) applied 1:50 (14). If not indicated otherwise cells were incubated with the mediators for 24h alone or in combination. When combinations of different reagents were used, they were applied simultaneously.

### Animal Experiments

After approval by the animal care committee (Laboratory Animal Care and Use Committee of the District of Unterfranken; AZ 2-272), experiments were performed in male C57BL6/J mice (Janvier Labs, Le Genest Saint Isle, France). Animals were kept under conditions that complied with the NIH *Guide for the Care and Use of Laboratory Animals*, and studies were approved by the governments of Unterfranken and Germany. Animals were kept on a standard diet and 12-hour day and night cycles.

### Experimental Setup

We used dextran sulfate sodium (DSS)-induced colitis in mice as (a murine) model for inflammatory bowel disease. Eight-week-old male mice received 2.5% DSS in autoclaved drinking water ad libitum for 4 days. The control group received normal drinking water *ad libitum*. Mice were monitored daily to evaluate the disease activity index (DAI) as described previously (8). Animals were randomized in different groups. One group received intraperitoneally (i.p.) 10 µM TP dissolved in 100µl 0.9% NaCl (DSS+TP) whereas the other group received 100µl 0.9% NaCl i.p. (DSS group). Injections were administered daily up from day 4 onwards every 24 hours for 3 days. Endpoint of the experiment was defined after 6 days.

### Measurement of Intestinal Permeability, Colon Length, and Tissue Harvesting

Intestinal permeability was measured as described previously (8): After 6 days, mice were anaesthetized mice using isoflurane (Forene, Abbott, Wiesbaden, Germany). Following laparotomy, the colon was mobilized and opened at the ileocaecal valve and at the upper rectum. After flushing the colon with PBS at RT to remove blood and stool, the colon was ligated at the cutted ends without compromising the blood supply. To determine intestinal permeability 200µl of 4kDa FITC dextran diluted in PBS (1 mg/ml) was injected in the ligated colon. After 1h blood from the inferior vena cava was taken to measure the concentration of 4 kDa FITC dextran translocated from the colonic lumen into the blood. The blood samples were centrifuged at 13.400 rpm for 10 minutes at 4°C, and the luminescence of the serum was quantified by using Genios Pro Reader (Tecan, Maennedorf, Switzerland). After blood collection mice were euthanized by exsanguination, the colon was harvested and the length was measured.

The ligations of the colon were removed and the colon was cut longitudinally into two pieces. One part was fixed in 4% paraformaldehyde embedded in paraffin and sectioned. The other half of the colon was lyzed and homogenized with a Tissue Lyzer (Quiagen, Hilden, Germany) in a SDS lysis buffer and used for Western Blot analysis.

### Histological Injury Score

Two blinded investigators quantified the inflammation of the tissue in H.E.-stained sections of the colon using the following inflammation score (26): Extent of inflammatory cell infiltration (none =1, mucosal infiltration =2, submucosal infiltration =3 transmural infiltration =4) and severity of epithelial damage (no epithelial damage =1, focal lesions =2, multiple lesions =3, extended ulcerations =4), resulting in a total scoring range from 2 – 8 per mouse.

### Immunostaining

Animal tissue samples were embedded in paraffin and sectioned in 1µm slices. Immunostaining was performed after removal of paraffin as described for CaCo2 cells.

Cultured cell monolayers were prepared for immunostaining as described previously (27). In brief, CaCo2 cells were grown to confluence on coverslips. After incubation with or without different mediators, cells were fixated with 2% formaldehyde for 10 minutes and permeabilized with 0.1% Triton-X100 for 15 minutes afterwards, at room temperature. The coverslips were incubated at 4°C overnight using following primary antibodies at 1:100 in phosphate-buffered saline (PBS): rabbit anti-Desmoglein2 (MyBiosource, Kampenhout, Belgium); mouse anti-Claudin2 (ThermoFisher, Schwerte, Germany); mouse anti-PI-3-kinase (Santa Cruz, Heidelberg, Germany). As secondary antibodies, we used Cy3- or 488- labeled goat anti-mouse, goat anti-rabbit (all diluted 1:600, Dianova, Hamburg, Germany). Coverslips were mounted on glass slides with Vector Shield Mounting Medium as anti-fading compound, which included DAPI to visualize cell nuclei additionally (Vector Laboratories, Burlingham, CA). Representative experiments were documented with a confocal microscope (Leica LSM 780) (Zeiss, Oberkochen, Germany).

### Western Blot

For Western blot analyses of mouse tissue, the specimens were lyzed in a SDS lysis buffer using TissueLyzer (Quiagen, Hilden Germany). CaCo2 cells were grown on 6-well plates, incubated with different mediators for 24h and finally homogenized in sodium dodecyl sulfate (SDS) lysis buffer containing 25 mmol/l HEPES, 2 mmol/l EDTA, 25 mmol/l NaF and 1% SDS. SDS gel electrophoresis and blotting were carried out after normalization of the protein amount using BCA assay (Thermo Fisher, Waltham, MA), as described previously (27). The following antibodies were used: mouse anti-Desmoglein 2 diluted 1:1000 (ThermoFisher, Schwerte, Germany); mouse anti-Claudin 2 diluted 1:700 (ThermoFisher, Schwerte, Germany); rabbit anti-PI-3-kinase diluted 1:500 (abcam, Cambridge, UK); rabbit anti-Phospho Akt (Ser473) diluted 1:1000 (Cell Signaling, Leiden, Netherlands); rabbit anti-Phospho Akt (Thr308) diluted 1:700 (Cell Signaling, Leiden, Netherlands), rabbit anti-Akt diluted 1:1000 (Cell signaling, Leiden, Netherlands) in 5% bovine serum albumin (BSA) and 0.1% Tween. As secondary antibodies horseradish peroxidase-labeled goat anti-rabbit IgG, goat anti-mouse IgG (all Santa Cruz Biotechnology, Heidelberg, Germany) were used (1:3000 in 5% BSA, 0.1% Tween). To validate equal loading of the gels peroxidase-labeled β-Actin or GAPDH (both Sigma-Aldrich, Munich, Germany) antibodies were applied. Chemiluminescence signal detection and quantification were performed by densitometry (ChemicDoc Touch Bio-Rad Laboratories GmbH, Munich, Germany). Optical densities (OD) were quantified in each Western Blot using Image Lab ChemicDoc Touch (Bio-Rad Laboratories GmbH, Munich, Germany) for statistical evaluation.

### Cell Culture

Caco2 cells (Caco2 Dsg2^WT^) were acquired from ATCC (Wesel, Germany) and were cultured in Eagle’s Minimum Essential Medium (EMEM, ATCC, Wesel, Germany) supplemented with 50 U/ml Penicillin-G, 50 µg Streptomycin and 10% fetal calf serum (FCS, Biochrom, Berlin, Germany). Cultures were used for experiments when grown to confluent monolayers. For experiments, cells were serum-starved for 24h. In addition, our Dsg2-deficient Caco2 cell line (Caco2 Dsg2^-/-^) was used as described in detail previously (8).

### Dispase-Based Enterocyte Dissociation Assays

As described previously (10), confluent cells in 24-well plates were exposed to the test reagents as indicated above, washed with Hank’s buffered saline solution plus (HBSS, Sigma-Aldrich, Munich, Germany) and incubated with Dispase-II (Sigma-Aldrich, Munich, Germany) for 30 minutes to release the monolayer from the well bottom. Afterwards, the cell sheet was exposed to shear stress by pipetting 5 times. Four fields of view were photographed with BZ-9000 (BIOREVO, Keyence, Osaka, Japan) and numbers were quantified.

### Measurement of FITC-Dextran Flux Across Monolayers of Cultured Epithelial Cells

As described previously (27), Caco2 cells were seeded on top of transwell filter chambers on 12-well plates (0.4μm pore size; Falcon, Heidelberg, Germany). After reaching confluence, cells were rinsed with PBS, and incubated with fresh DMEM without phenol red (Sigma) containing 10 mg/ml FITC-dextran (4 kDa). Paracellular flux was assessed by taking 100μl aliquots from the outer chamber over 2 h of incubation. Fluorescence was measured using a Tecan GENios Microplate Reader (MTX Lab systems, Bradenton, USA) with excitation and emission at 485 and 535 nm, respectively. For all experimental conditions, permeability coefficients (P_E_) were calculated by the following formula (28): P_E_ = [(ΔC_A_/Δt) × V_A_]/S × ΔC_L_, where P_E_ = diffusive permeability (cm/s), ΔC_A_ = change of FITC-dextran concentration, Δt = change of time, V_A_ = volume of the abluminal medium, S = surface area, and ΔC_L_ = constant luminal concentration. Experiments were performed in triplicates for each conditions and mean values of each triplicates were taken together as one independent experiment.

### Measurements of Transepithelial Electrical Resistance (TER)

To measure transepithelial electrical resistance (TER) we used ECIS trans-Filter Adapter for ECIS 1600R across cell monolayers (Applied Biophysics, Ibidi GmBH, Martinsried, Germany). Cells were seeded on 8 well arrays with 40 electrodes per well (Applied Biophysics, Ibidi GmBH, Martinsried, Germany) as described previously (8). At confluency of monolayers cells were treated with different mediators and measurements were started immediately after application of the reagents.

### Real Time Quantitative (q)RT-PCR

RNA from CaCo2 wildtype and CaCo2 Dsg2 knock-out cells was isolated using TRIZOL and cDNA was synthesized with iScript™ cDNA Synthesis Kit (Biorad, Munich, Germany). Quantitative PCR was performed using MESA GREEN qPCR MasterMix Plus for SYBR^®^ Assay No ROX (Eurogentec, Cologne, Germany) on the CFX96 Touch Real-Time PCR Detection System (Biorad, Munich, Germany). Gene expression was analyzed *via* the Bio-Rad CFX Manager software with β-actin as a reference gene. All reactions were done in duplicates at 60.0°C annealing temperature. Primers were applied at a concentration of 5 µM. Primer sequences: humanDSG2 f: 5`- AACGACAACTGTCCCACACT -3`, humanDSG2 r: 5`- TTTCTTGGCGTGCTATTTTC -3`; human claudin2 f: 5`- CTCCCTGGCCTGCATTATCTC -3`; human claudin2 r: 5`- ACCTGCTACCGCCACTCTGT -3`

### Membrane Protein Extraction Assay

Protein fractionation was carried out using Mem-Per Plus Kit (Thermo Fischer, Waltham, MA, USA) as described previously (8). Cells were harvested in growth media by scraping them from the bottom with a cell scraper. After centrifugation at 300 rpm for 5 minutes and washing three times, cells were permeabilized with a permeabilization buffer to release the cytosolic fraction. The cytosolic fraction was separated by centrifugation at 16.000 rpm for 15 minutes. The pellet containing the membrane-associated proteins was then re-suspended in a solubilization buffer. The suspension was centrifuged another time at 16.000 rpm for 15 minutes to remove particulate material. Then the cytosolic and membrane-associated supernatants were used for Western Blot analysis.

### Co-Immunoprecipitation Experiments

Cells were seeded on 6-well plates. Monolayer cells at confluency were treated with different mediators and harvested after different incubation times in RIPA-buffer (ThermoFisher). Samples were steamed for 1min and centrifuged for 15min at 15.000g and 4°C. Total protein concentration was determined by measuring absorbance at 280nm.

The Co-IP experiments were done using the immuno-precipitation Starter Pack (GE Healthcare, Germany). The amount of 300-600 µg protein was used. After an initial pre-clearing step of one hour at 4°C (500µl of whole cell lysate with respectively 25µl protein G/A sepharose beads), antigens were coupled overnight at 4°C to 2.5 µg purified antibody rabbit anti-PI-3-kinase (Santa Cruz Biotechnology, Heidelberg, Germany). Protein-antibody complexes were precipitated with a mix of 25µl protein A and G sepharose beads for one hour at 4°C. The beads were washed three times with isotonic salt buffer (RIPA-buffer), once with wash-buffer (50 mM TRIS, pH 8) and suspended in 50µl Laemmli buffer. After denaturation for 5min at 95°C and a following centrifugation step, the supernatant was analyzed by western Blot analyses as described above. Detection was performed with mouse anti-Dsg2 diluted 1:1000 (Invitrogen, Carlsbad, CA, USA). Optical densities (OD) were quantified in each Western Blot using Image Lab ChemicDoc Touch (Bio-Rad Laboratories GmbH, Munich, Germany) for statistical evaluation. A ratio of the co-immununoprecipitated proteins was calculated to determine the potential changes detected under different conditions.

### Proximity Ligation Assay

Proximity ligation assays (PLA) were carried out as recommended by the manufacturer ((Sigma-Aldrich, Munich, Germany): In brief, cells were seeded on coverslips and treated with TNFα for 24h when reached confluency. Two primary antibodies from different species were selected. Following antibodies were used: mouse anti-Dsg2 (Invitrogen, Carlsbad, CA, USA) at a dilution of 1:100, rabbit anti-PI-3-kinase 1:100 (Cell signaling, Leiden, Netherlands), mouse anti-Plakoglobin 1:100 (Progen, Heidelberg, Germany) and rabbit anti-Dsg2 1:100 (abcam, Cambrige, UK). After blocking of unspecific binding sites, slides were incubated with the mentioned primary antibodies. Next, a pair of oligonucleotide-labeled secondary antibodies (PLUS and MINUS Probes) which bind to the primary antibody were applied. When the PLA probes are in close proximity, connector oligos join the PLA probes and become ligated by addition of ligase at a dilution of 1:40. As a consequence, a closed circle DNA template is formed and acts as a primer for a DNA polymerase. Finally, labeled oligos hybridize to the complementary sequences within the amplicon, which are then visualized as discrete spots (PLA signals) by microscopy analysis. As negative controls, the same procedure was carried without application of primary antibodies as recommended by the manufacturer.

### Quantification of Immunostaining

Quantification of immunostaining was carried out as described previously (9). In brief, 10μm line was placed orthogonal to the cell border with the cell border representing the middle of this line. The fluorescence pixel intensity was measured using ImageJ, which resulted in a graph with a maximum peak in the middle of the curve if the staining pattern was at the cell border. Loss of staining intensity at the cell borders resulted in a flattening of the curve. Indicating a redistribution of the proteins in the cytoplasm. For each sample at least ten randomly chosen junctions were measured by a blinded observer. For merge images the position and length of each line was then saved and also measured in the corresponding image field, thus resulting in 60 measurements at 30 distinct junctions per condition. After calculating the average and subtracting the gray value of the backgrounds three lane profiles per protein and condition were analyzed using a Two-way ANOWA with multiple comparisons.

### Statistics

Statistical analysis was performed using Prism (GraphPad Software, La Jolla, CA, USA). Data are presented as means ± SE. Statistical significance was assumed for p<0.05. Paired Student’s t-test was performed for two-sample group analysis after checking for a Gaussian distribution. Analysis of variance (ANOVA) followed by Tukey’s multiple comparisons test and Bonferroni correction was used for multiple sample groups. The tests applied for each of the different experiments are indicated in the figure legends.

## Results

### DSS-Induced Colitis and Increased Intestinal Permeability in Mice Were Attenuated by Application of Dsg-Linking Tandem Peptide (TP)

Previously, we demonstrated that Dsg-linking tandem peptide (TP) restored TNFα-induced loss of intestinal epithelial barrier function in Caco2 cells *in vitro* by increasing Dsg2-mediated adhesion (10). To test the effects of TP on IEB in an *in vivo* model of intestinal inflammation, we induced acute colonic injury with 2.5% dextran sulfate sodium (DSS) in mice. C57Bl/6 mice (n=17) received 2.5% DSS (n=6) in autoclaved drinking water ad libitum, whereas control mice (n=5) received normal drinking water. One group of mice (DSS + TP, n=6) were injected intraperitoneally beginning at day 4 with 10 µM of TP in 100µl 0.9% sodium chloride whereas the other group (DSS) received 100µl 0.9% sodium chloride i.p daily.

**Figure 1:**
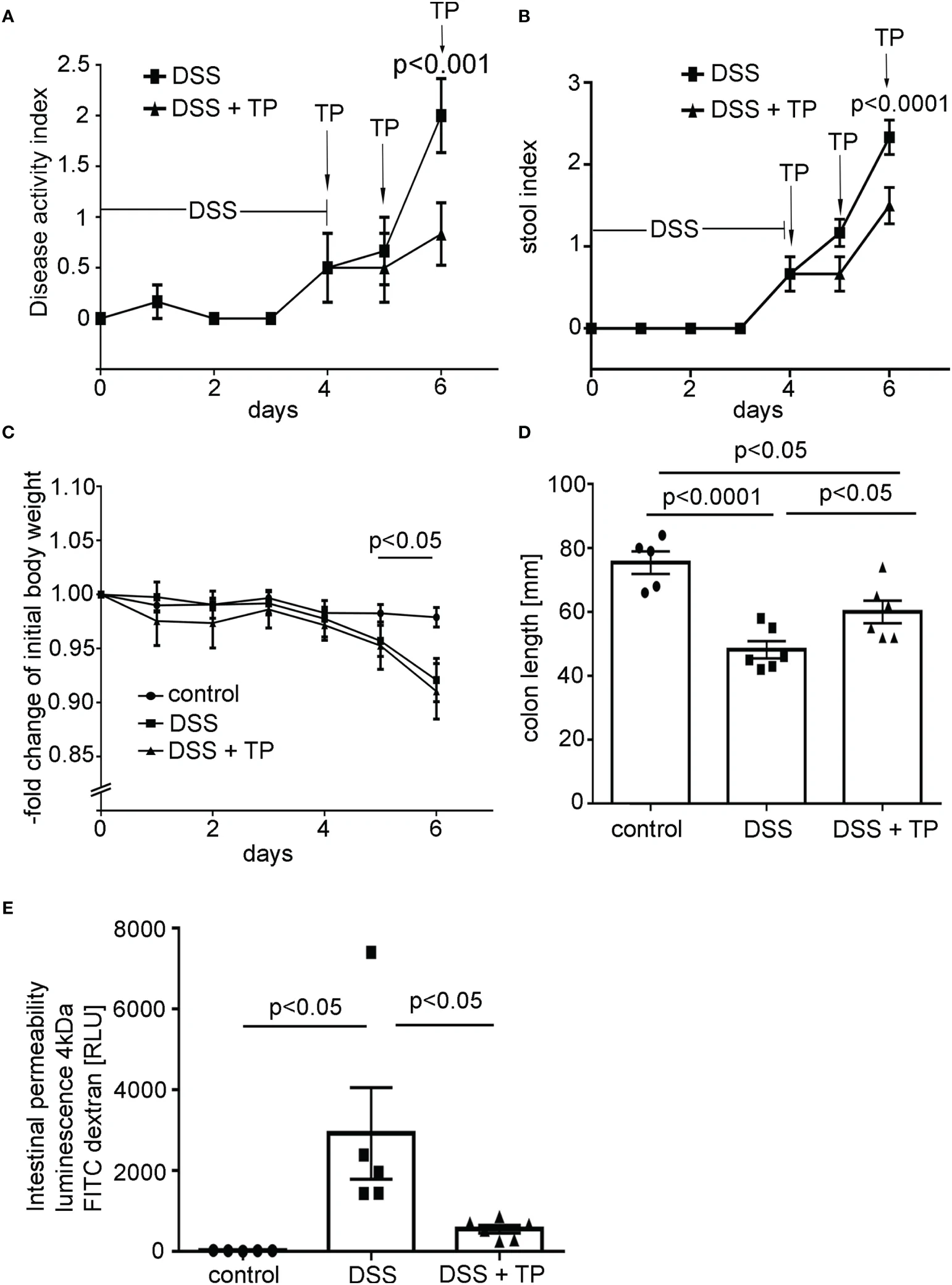
DSS- induced colitis in mice was attenuated by application of Tandem peptide (TP). **(A)** Disease activity index (DAI) is shown for C57/Bl6 mice receiving 2.5% DSS alone or in combination with TP at 10 µM i.p. (n= 6) for each group. DAI was significantly reduced in mice treated with TP (p<0.0001; unpaired t-test for each time point). **(B)** The stool index was increased in DSS colitis and significantly reduced (p<0.0001) at day 6 in mice treated with TP (unpaired t-test for each time point). **(C)** Body weight of control animals was unchanged in the course of experiments whereas in mice with DSS colitis body weight was significantly reduced (p<0.05; unpaired t-test for each time point). **(D)** DSS treatment resulted in a significantly reduced colon length (p<0.0001, n=8) compared to controls. Treatment with TP attenuated colon length reduction compared to DSS animals (p<0.05, n=8) (ordinary 1-way ANOVA). **(E)** Measurements of 4 kDa FITC dextran flux across the IEB show increased levels of luminescence in the blood compared to controls (n=5, p<0.05). Treatment of DSS animals with TP attenuated increased intestinal permeability compared to DSS induced colitis (n=5, p<0.05) (ordinary one-way ANOVA).

**Figure 2:**
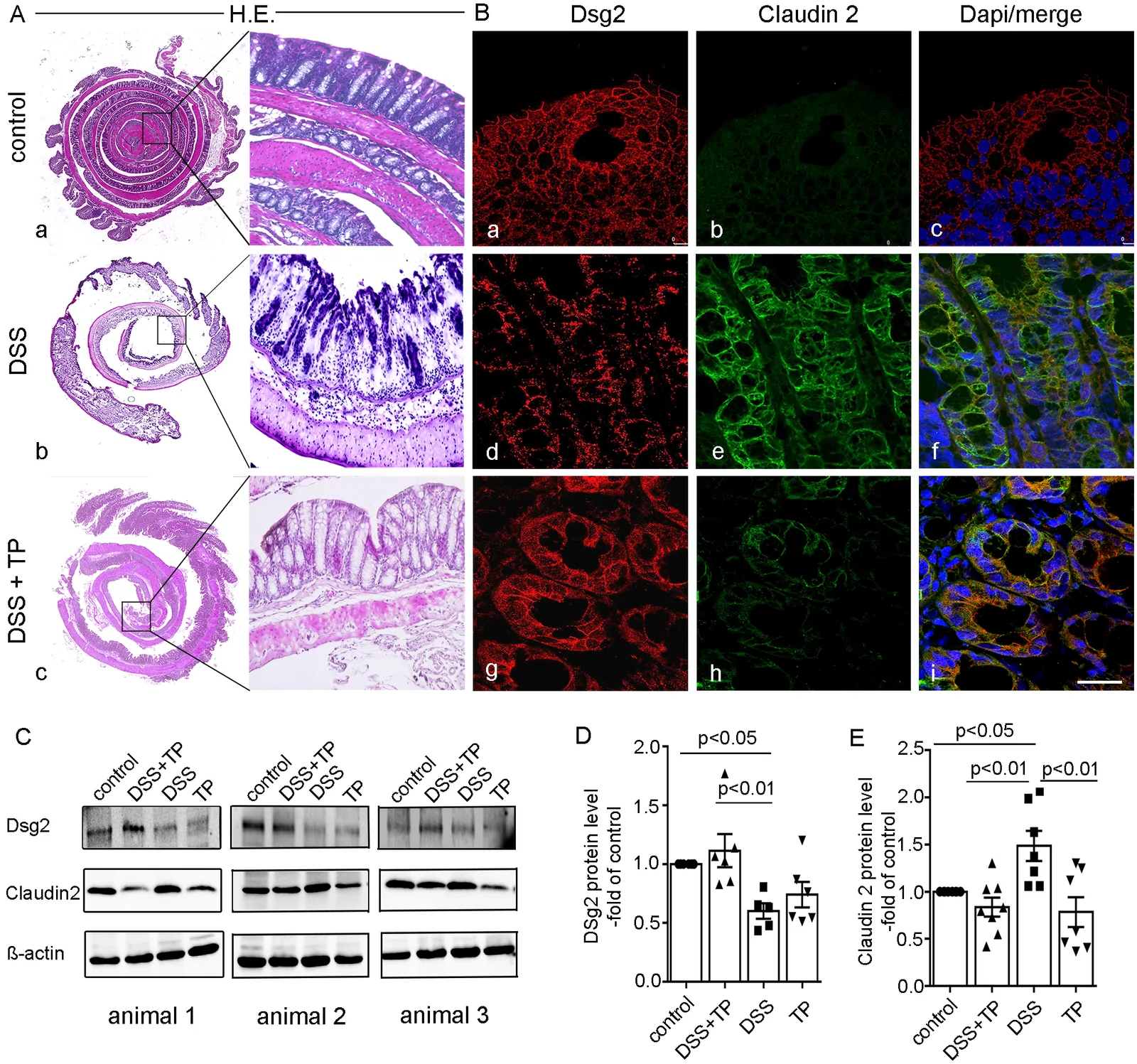
TP affected DSS-induced redistribution of Dsg2 and Cld2 at the cell border *in vivo*. **(A)** Representative H&E staining of mouse colon are shown (left overview; right magnification). Controls (a) show a normal morphology of the gut whereas DSS (2.5% DSS) animals (b) show signs of severe inflammation with mucosal damage. Treatment of animals with 10 µM TP (c; DSS+TP) attenuated inflammation-induced changes in the colon. Images shown are representatives for n=6 in each group. **(B)** Immunostaining for Dsg2 (a, d, g), Claudin2 (b, e, h) or merged images with staining of cell nuclei using DAPI (c, f, i) are shown for the same conditions. The loss of Dsg2 in DSS animals (d) compared to controls (a) is attenuated by TP treatment (g). This is paralleled by augmented Claudin2 staining (e) in DSS colitis compared to controls (b) whereas TP blocked inflammation-induced increase of Claudin2 (h). Images shown are representatives of n=6 in each group. Scale bar is 25 µm. **(C)** Western Blots from colon lysates of control animals, animals with DSS colitis treated with TP (DSS+TP), DSS colitis alone and with TP alone are shown for Dsg2 and Claudin2 and β-actin. A set of 3 different animals is shown. **(D)** Quantitative analyses of the optical densities for Dsg2 Western blot bands normalized to β-actin showed reduced Dsg2 compared to DSS-colitis. This reduction was not observed in DSS+TP-treated animals (p<0.05 compared to the control group, n=6; p<0.01 compared to DSS+TP-treated mice; TP showed no significant change compared to all other groups; n=6 for each group, ordinary 1-way ANOVA). **(E)** Quantitative analyses of the optical densities for Claudin2 Western Blot bands normalized to β-actin showed significantly increase of Claudin2 in DSS mice compared to control mice (p<0.05) which was blocked by TP (p<0.01) (n=6 for each group; ordinary one-way ANOVA).

Following DSS application, the disease activity index (DAI) and the stool index were increased to 2.0 ± 0.75 and 2.33 ± 0.89 (**Figures 1A, B**). Both were significantly reduced at day 6 (endpoint of the experiment) in animals treated with TP. The bodyweight was reduced in the DSS group compared to controls and following treatment with TP (**Figure 1C**). In the control group, the colon length was 75.4 ± 3.54mm which was reduced to 48.17 ± 2.72mm in the DSS group. Reduction of colon length was attenuated by TP application (60.0 ± 3.55mm; **Figure 1D**). Intestinal permeability as revealed by measurements of 4 kDa FITC–dextran flux across the IEB was increased in DSS animals compared to control animals whereas treatment with TP attenuated inflammation-induced increase of intestinal permeability (**Figure 1E**). Histological analyses of H&E-stained colon sections showed a severe inflammation pattern after treatment with DSS (**Figure 2Ab**) which was attenuated by injection of TP (**Figure 2Ac**). TP application alone did not have an obvious effect on crypt architecture in colon sections (data not shown). Histological injury score also revealed an acute inflammation following DSS administration (control 2.00 ± 0.01 *vs.* DSS 7.13 ± 0.48, p<0.0001). Again, TP reduced inflammation (histological score DSS+TP 5.63 ± 0.52, p<0.05 compared to DSS) (data not shown).

To verify the close correlation between the integrity of Dsg2 and Claudin2 that we had previously observed *in vitro* (10), immunostaining was carried out for both proteins from the gut specimens of the different experimental groups of the *in vivo* experiments. In the control group, Dsg2 in colon sections showed a regular staining pattern along the cell borders of enterocytes whereas Claudin2 was hardly detectable (**Figure 2Ba–c**). In DSS-colitis, Dsg2 was reduced in intestinal epithelium whereas Claudin2 was present along virtually all enterocytes (**Figure 2Bd–f**). Both the reduction of Dsg2 and the strong increase of Claudin2 were attenuated when TP was applied to animals with DSS-colitis (**Figure 2Bg–i**). In line with this, in Western blot analyses DSS-induced reduction of Dsg2 compared to control conditions (p<0.05, n=6) was restored after TP treatment (p<0.01, n=6) (**Figures 2C, D**). Moreover, DSS-induced increase of Claudin2 compared to controls (p<0.05, n=6) was decreased after TP application (p<0.01, n=6) (**Figure 2E**). In summary, these data confirmed a critical role of Dsg2 for intestinal barrier stabilization and accordingly TP is effective to stabilize intestinal barrier function *in vivo*. Additionally, the close correlation between the integrity of Dsg2 and Claudin2 strengthened our hypothesis of a causal interaction between both proteins in the regulation of intestinal permeability.

### TNFα-Induced Loss of Dsg2-Mediated Adhesion Augmented Claudin2 Expression in Caco2 Monolayers

**Figure 3:**
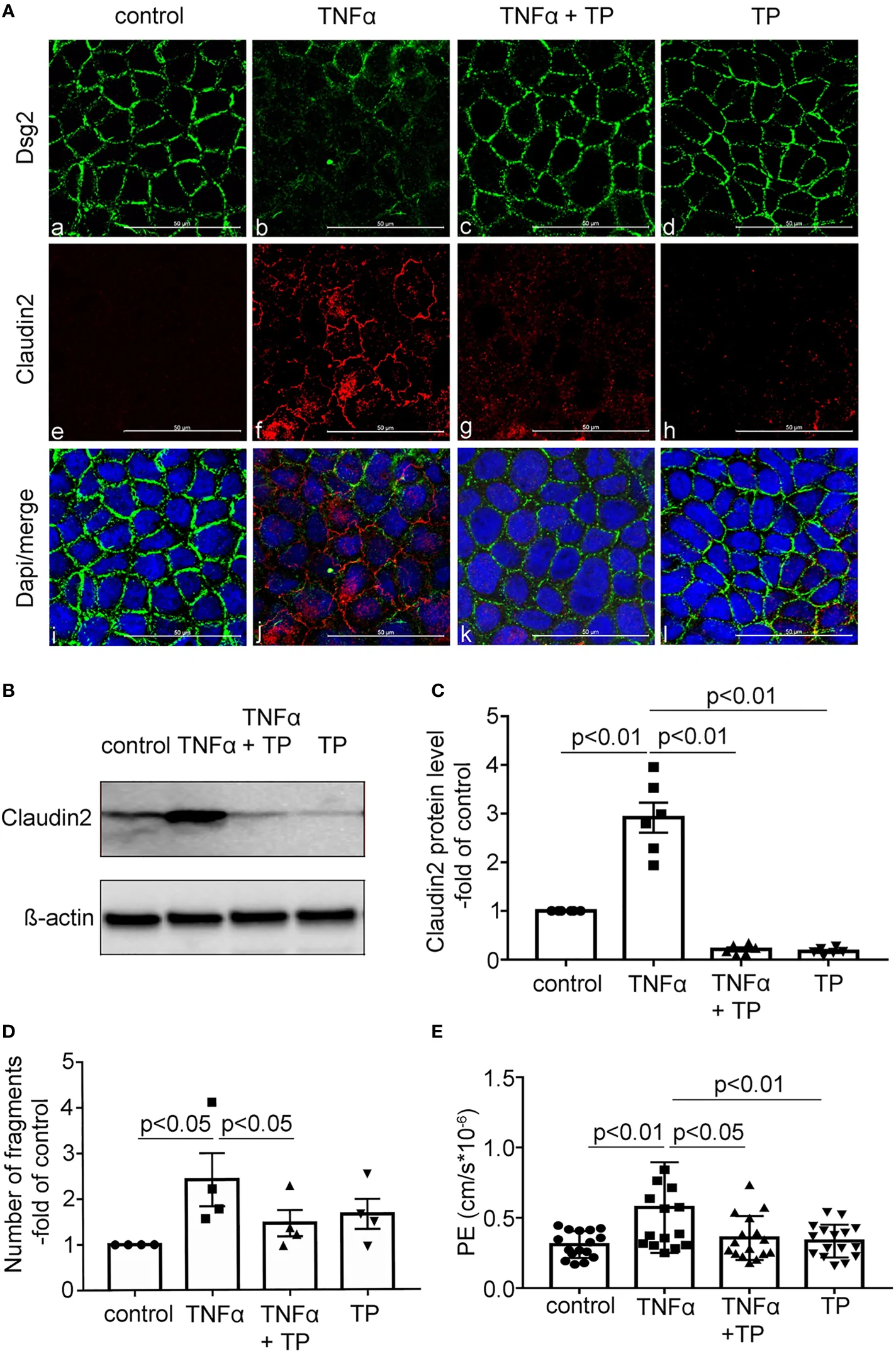
TP attenuated TNFα-induced redistribution of Dsg2 and Cld2 *in vitro*. **(A)** Representative immunofluorescence staining of Caco2 cells after 24h treatment with either TNFα (100 ng/ml) or TP (20 µM) or both (TNFα+TP) are shown for Dsg2 (green; a-d), Claudin2 (red; e-h) or merged images with DAPI staining (blue; i-l) to visualize cell nuclei. Compared to controls (a,e,i) application of TNFα resulted in reduced Dsg2 (b) and increased Claudin2 (f) at the cell borders. TNFα-induced changes were blunted by application of TP (c,g,k). TP alone had no effect on Dsg2 and Claudin2 (d,h,l) compared to controls. Images shown are representative for n=7, scale bar is 50 µm. **(B)** Representative Western Blot from Caco2 lysates under the conditions outlined in A for Claudin 2 is shown; ß-actin is shown as loading control. **(C)** Quantitative analyses of the optical densities for Claudin2 Western Blot bands normalized to β-actin showed a significant increase of Claudin2 protein levels compared to controls which is attenuated following incubation of cells with TNFα+TP. TP alone has no effect on Claudin2 expression. N= 4 for controls and n=6 for all other experimental conditions; ordinary 1-way ANOVA **(D)** Results from enterocyte dissociation assays are shown for the same conditions outlined in **(A)** Incubation of Caco2 cells with TNFα results in increased fragmentation of monolayers whereas TNFα+TP blocked this effect. Effects of TP alone on Caco2 monolayers were absent (p<0.05, n=4, ordinary 1-way ANOVA). **(E)** Permeability coefficient (P_E_) measured as 4 kDa FITC dextran flux across confluent Caco2 monolayers are shown. TNFα-induced increase of epithelial permeability compared to controls (p<0.01) was blocked by application of TP (p<0.05, compared to TNFα); n=16 for each group; ordinary 1-way ANOVA.

Next, we used our *in vitro* system of differentiated Caco2 cells and performed immunostaining following TNFα application to mimic inflammation. In Caco2 cells, TNFα resulted in reduced Dsg2 at the cell borders compared to untreated controls (**Figure 3A**). TNFα-induced loss of Dsg2 was completely blocked when TP was applied while TP application alone did not have an effect on Dsg2 staining (**Figure 3Ac, d**). Claudin2 staining was augmented when TNFα was applied to epithelial monolayers (**Figure 3Ae, f**), which was attenuated following TP application (**Figure 3Ag, h**). Western blot analyses confirmed a strong increase of Claudin2 expression in Caco2 cells following application of TNFα, which was blocked even below control levels when TNFα and TP were applied together (**Figure 3Ag, h, B, C**). Dsg2 expression did not change significantly in Western blot analysis under the different conditions (data not shown). However, in line with the observed loss of Dsg2 at the cell borders, dispase-based enterocyte dissociation assays demonstrated that TNFα led to a 2.5-fold increased fragmentation of epithelial monolayers (**Figure 3D**). Application of TP blocked TNFα-induced cell dissociation which confirmed the significant role of Dsg2 in this context (**Figure 3D**) (n=4, p<0.05). Measurements of 4kDa FITC dextran flux across CaCo2 monolayers showed augmented epithelial permeability following incubation with TNFα (**Figure 3E**) (n=16, p<0.01). This was attenuated when TP was applied together with TNFα (n=16, p<0.05). Taken together, the *in vitro* experiments in CaCo2 monolayers confirmed that TNFα-induced loss of Dsg2 at the cell borders led to increased epithelial permeability, cell dissociation and Claudin2 expression. All of these effects were attenuated when Dsg-stabilising TP was applied, suggesting a direct link between Dsg2 integrity and Claudin2 expression.

### Loss of Dsg2 Integrity Increased Claudin2 Expression

**Figure 4:**
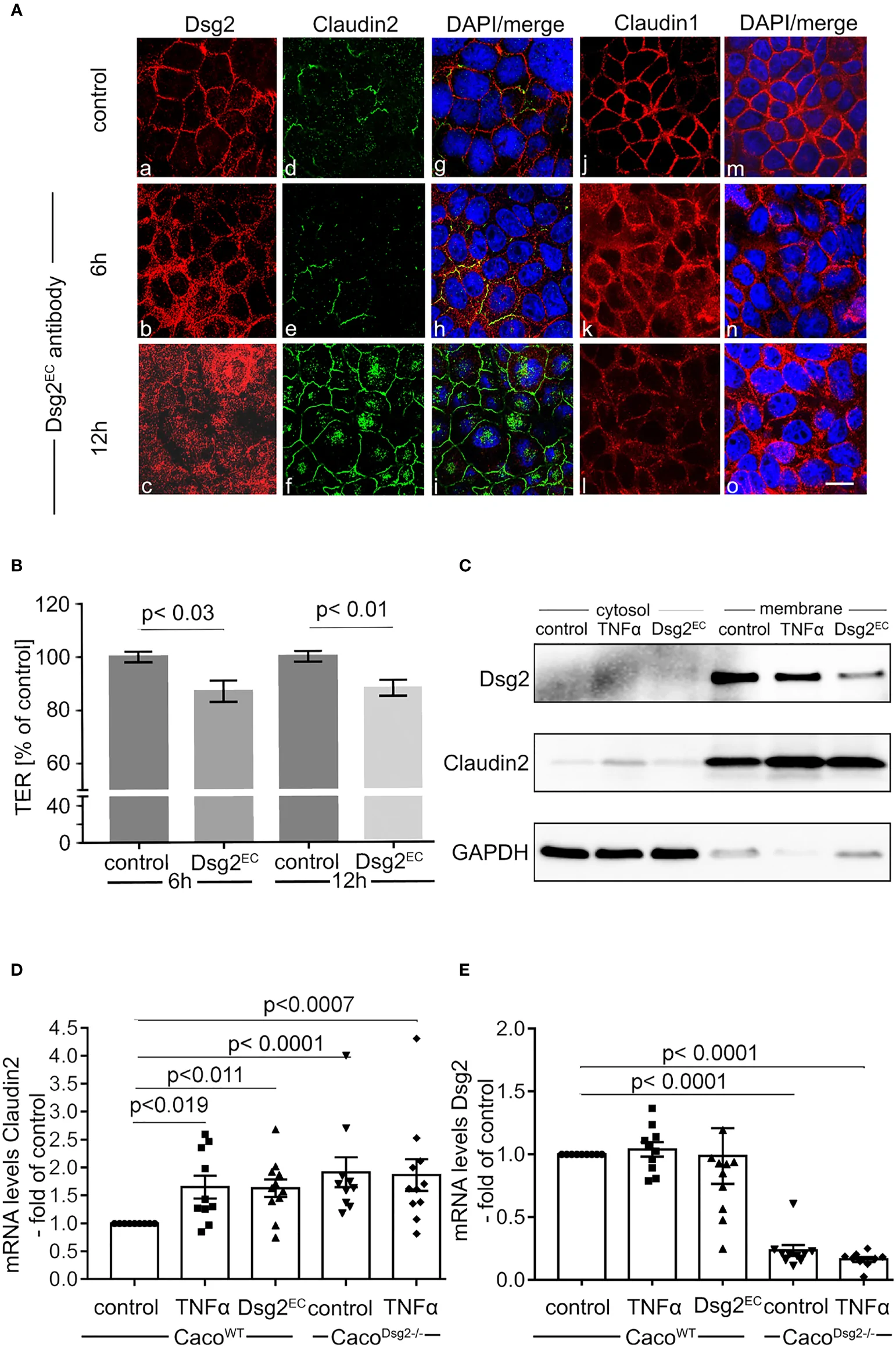
Antibody-induced loss of Dsg2 increased Claudin2 at the cell borders.**(A)** Immunostaining of Caco2 monolayers for Dsg2 (a-c) Claudin2 (d-f), Claudin1 (j-l) following incubation with a Dsg2 antibody directed against parts of the extracellular domain of Dsg2 (Dsg2^EC;^ 1:50) for 6h (b, e, h, k, n) and 12h (c, f, i, o) are shown. Application of Dsg2^EC^ results in loss of Dsg2 at the cell borders (b,c) compared to controls (a). This was paralleled by increased Claudin2 (e,f) whereas changes of Claudin1 staining following Dsg2^EC^ application are modest (k,l). Images are representative for n=8; scale bar is 20 µm. **(B)** Application of Dsg2^EC^ antibody decreases Transepithelial Electrical Resistance (TER) after 6h (p<0.03, n=14, ordinary 1-way ANOVA) and 12h (p<0.01, n=14, ordinary 1-way ANOVA) and 12h compared to untreated CaCo2 cells .**(C)** Representative Western Blot after cell compartment separation assay is shown for Dsg2 and Claudin2 after incubation of Caco2 with TNFα (24h) or Dsg2^EC^ (12h). GAPDH served to document the separation in cytosolic and membrane fraction. Both TNFα and Dsg2^EC^ decrease Dsg2 and augment Claudin2 the membrane-bound fraction; n=5. **(D)** Quantitative (q)RT-PCR showed that Claudin2 expression was elevated in Dsg2-deficient Caco2 cells (Dsg2^-/-^) under basal conditions compared to CaCo2^WT^ (p<0.0001, n=10, ordinary 1-way ANOVA). Application of TNFα (24h) increased Claudin2 mRNA levels in Caco2^WT^ compared to untreated cells (p<0.019, n=10, ordinary 1-way ANOVA) but not in CaCo2 ^Dsg2-/-^. **(E)** qRT-PCR shows that mRNA levels of Dsg2 are not altered following application of Dsg2^EC^ antibody and no Dsg2 was detectable in Caco2 Dsg2^-/-^ (n=10, ordinary 1-way ANOVA).

**Figure 5:**
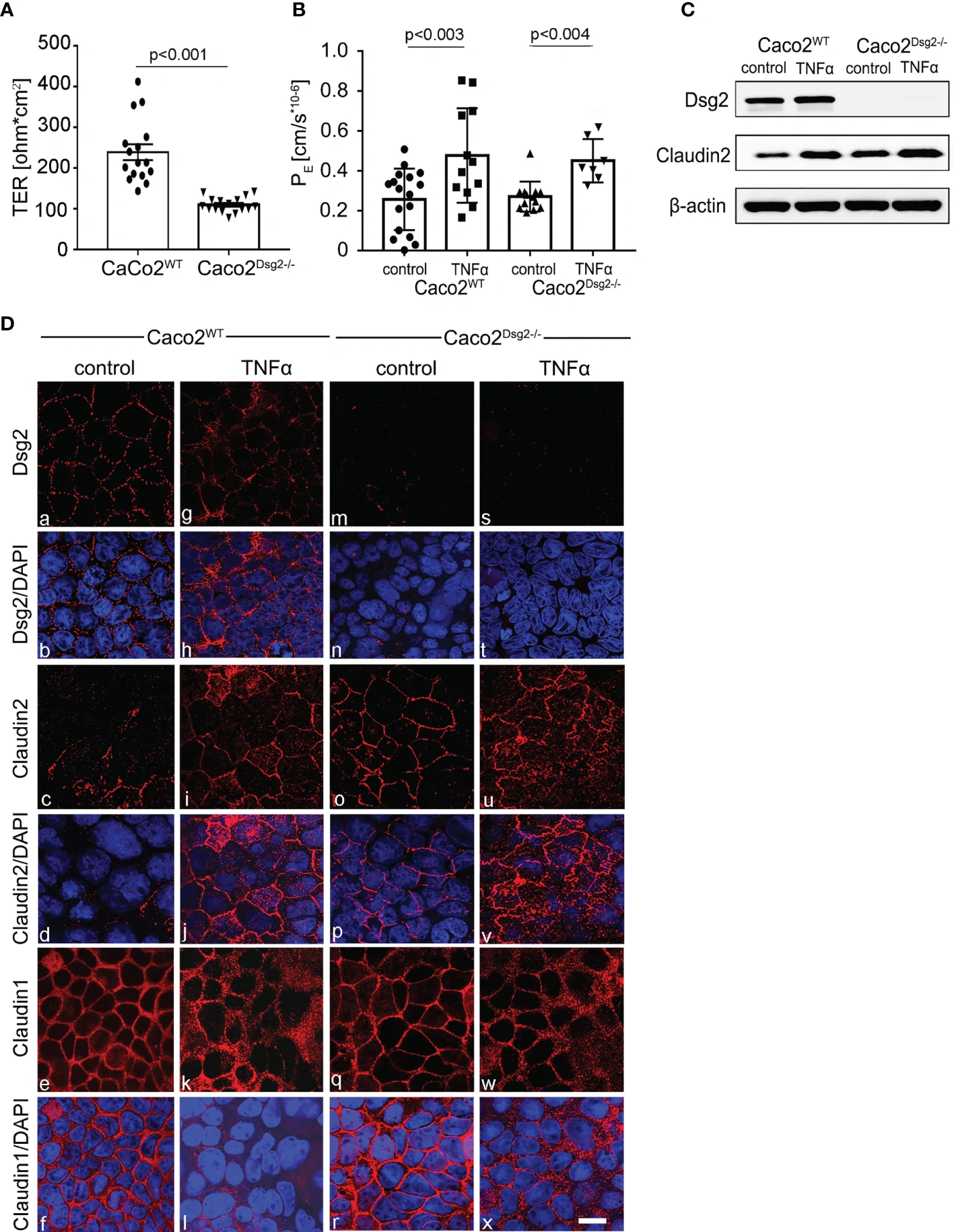
Claudin2 was increased following Dsg2 knockout in Caco2 cells. **(A)** Measurements of Transepithelial Electrical Resistance (TER) of CaCo2^WT^ compared to CaCo2 Dsg2^-/-^ monolayers under baseline conditions demonstrate reduced baseline TER in Dsg2-deficient cells (p<0.001, n=13, ordinary 1-way ANOVA). **(B)** Permeability coefficient (P_E_) of 4kDa FITC Dextran on Caco2^WT^ cells (p<0.003 compared to control, n=12, ordinary 1-way ANOVA) and in Caco2 Dsg2^-/-^ cells (p<0.004 compared to Caco2 Dsg2^-/-^ control, n=12, ordinary 1-way ANOVA) show increased permeability after incubation with TNFα whereas no differences under baseline conditions are obvious (n=12, ordinary 1way ANOVA). **(C)** Western Blot experiments of CaCo2^WT^ and CaCo2 Dsg2^-/-^ monolayers is shown for Dsg2, Claudin2 and for ß-actin to verify equal loading of the Western Blots. This confirms loss of Dsg2 in CaCo2 Dsg2^-/-^ cells and augmented basal expression of Claudin2 compared to CaCo2^WT^; the experiment shown is representative for n=8. **(D)** Immunostaining of Caco2^WT^ and Caco2 Dsg2^-/-^ for Dsg2 (a, g, m, s), Claudin2 (c, i, o, u) and Claudin1 (e, k, q, w) are shown under baseline (control) conditions (a-f, m-r) and following application of TNFα (g-l, s-x) for 24h. Dsg2 is found at the cells borders in Caco2^WT^ under control conditions (a). TNFα leads to a loss of Dsg2 at the cell borders (g) and in Caco2 Dsg2 ^-/-^ no Dsg2 was detectable (m, s). Claudin2 is hardly detectable in Caco^WT^ under control conditions (c) but can be found at the cell membrane after TNFα incubation (i). In Caco2 Dsg2^-/-^ cells Claudin2 staining pattern shows a distribution at the cell borders in both control and after TNFα treatment (o, u). Claudin1 is found at the membrane in both Caco2WT and Caco2Dsg2-/- cells under control conditions (e, q), which is reduced following application of TNFα (k, w) (n=4; scale bar is 20 µm).

To directly interfere with Dsg2-mediated adhesion, we applied a monoclonal antibody directed against the extracellular second and third extracellular repeat domains of Dsg2 (anti-Dsg2^EC^) on Caco2 monolayers as described previously (6, 14). This resulted in a reduction of Dsg2 at the cell borders after 6h and 12h of incubation with anti-Dsg2^EC^ in immunostaining (**Figure 4Aa–c**). The loss of Dsg2 at the cell borders was paralleled with a strong increase of Claudin2 which was sparsely seen under control conditions and regularly present at the cell borders after 6h and after 12h of incubation with anti-Dsg2^EC^ (**Figure 4Ad–f**). In contrast, the staining pattern of barrier-sealing TJ protein Claudin1 was modestly changed when compared to Claudin2 staining pattern after 6h and 12h of incubation with anti-Dsg2 ^EC^ (**Figure 4Aj–l**). In line with this, application of anti-Dsg2^EC^ led to decreased Transepithelial Electrical Resistance (TER) after 6h and 12h to 0.87 ± 0.04-fold and 0.88 ± 0.03-fold of control (**Figure 4B**) whereas measurements of 4 kDa FITC dextran flux remained unaltered under these conditions (**Figure 5B**).

In cell compartment separation assays application of anti-Dsg2^EC^ or TNFα reduced Dsg2 in the membrane-bound fraction compared to controls (**Figure 4C**). This was paralleled with an increase of Claudin2 in the membrane-bound fraction both after application of anti-Dsg2^EC^ and after incubation with TNFα. In line with these observations, application of anti-Dsg2^EC^ and TNFα augmented Claudin2 mRNA-levels in Caco2 Dsg2^WT^ (**Figure 4D**) whereas Dsg2 mRNA levels were not affected (**Figure 4E**).

To further determine a causal relationship between loss of Dsg2 and increased Claudin2 we used our Dsg2-deficient Caco2 cell line (Caco2 Dsg2^-/-^) (8). Measurements of TER values across differentiated monolayers revealed a significantly higher basal resistance in Caco2^WT^ of 238 ± 20 Ω*cm² compared to Caco2 Dsg2^-/-^ with 110 ± 5 Ω*cm² when cultured under the exact same conditions (**Figure 5A**). However, Permeability coefficient-values as revealed by measurements of 4 kDa FITC dextran flux across epithelial monolayers showed that basal permeability was not different when Caco2^WT^ and Caco2 Dsg2^-/-^ were compared (**Figure 5B**). Incubation of monolayers with TNFα increased permeability in both cell lines (**Figure 5B**). In immunostaining of differentiated Caco2 Dsg2^WT^ monolayers Dsg2 was regularly distributed at the cell borders whereas Claudin2 was sparsely found (**Figure 5Da–d**). In Caco2 Dsg2^-/-^ no Dsg2 (**Figure 5Dm**) was detected in immunostaining whereas Claudin2 (**Figure 5Do**) was found to be regularly present at the cell borders under basal conditions. Application of TNFα resulted in reduced Dsg2 (**Figure 5Dg**) and significantly augmented Claudin2 staining at the cell borders in Caco2 Dsg2^WT^ (**Figure 5Di**). In Caco2 Dsg2^-/-^, Claudin2 was located at the cell borders under both conditions (untreated and TNFα) (**Figure 5Do, u**). Claudin1 was regularly distributed at the cell borders of Caco2 Dsg2^WT^ and Caco2 Dsg2^-/-^ under basal conditions (**Figure 5Do, u**) which was reduced by application of TNFα (**Figure 5Dk, w**).

In Western blot analyses Dsg2 was equally expressed under basal conditions and following application of TNFα in Caco2^WT^ (**Figure 5C**) whereas no Dsg2 was found in Caco2 Dsg2^-/-^ (**Figure 5C**). In WT cells Claudin2 was augmented following application of TNFα (**Figure 5C**). In Caco2 Dsg2^-/-^ basal expression of Claudin2 was increased compared to Caco2^WT^ and was further augmented after incubation with TNFα (**Figure 5C**).

In line with this, qRT-PCR showed similar results indicating changes of Claudin2 at the level of protein expression rather than on changes in protein turnover (**Figure 4D**).

### Loss of Dsg2 Augmented Basal Activation of PI-3/AKT-Signaling

**Figure 6:**
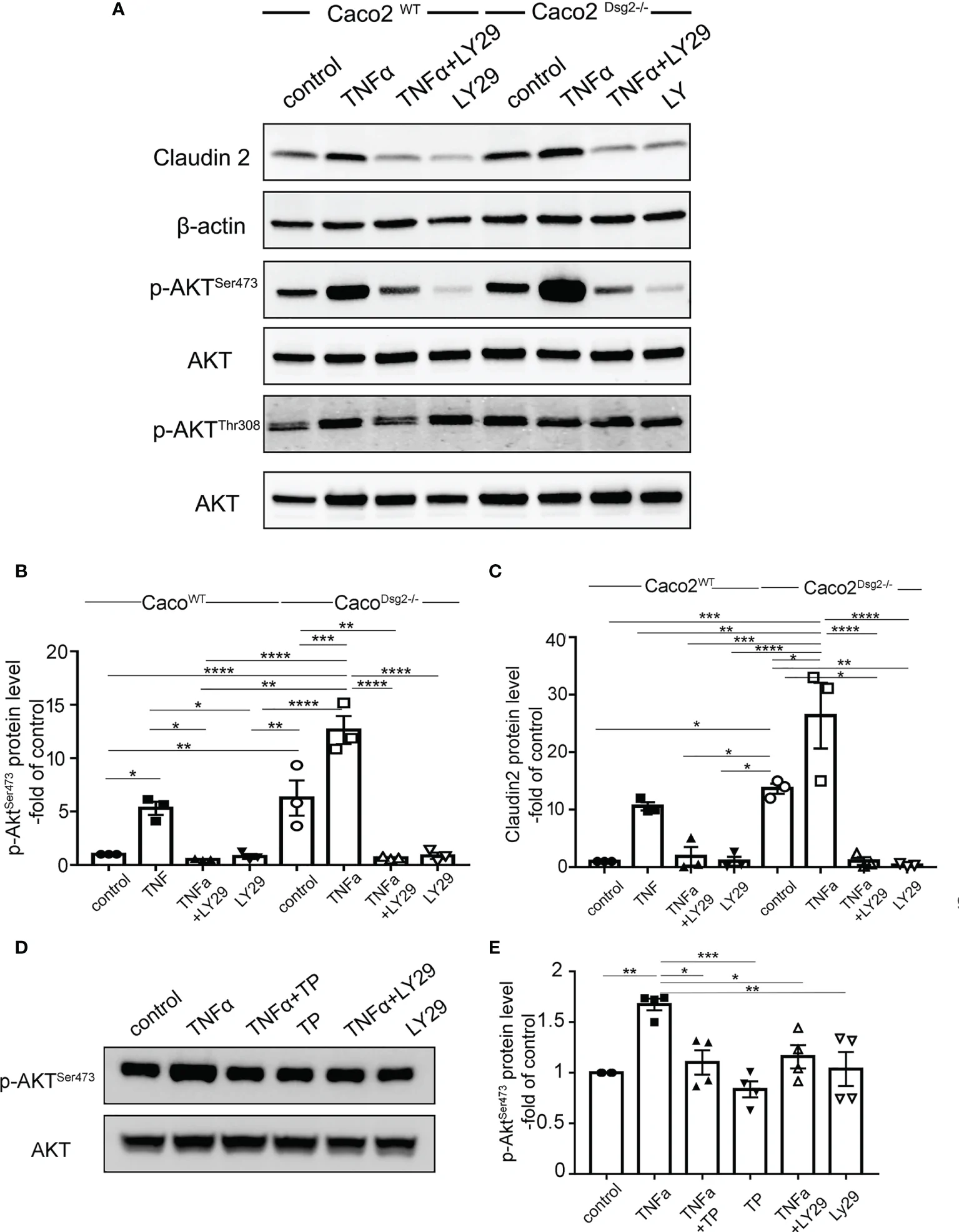
PI-3K/Akt pathway is involved in the regulation of Claudin2. **(A)** Western Blot of Caco2^WT^ and Caco2 Dsg^-/-^ are shown for Claudin2, p-AKT^Ser473,^p-AKT^Thr308^, total AKT and ß-actin as loading control under the different experimental conditions. Experiment shown is representative for n=3. **(B)** Quantitative analyses of the optical densities for p-AKTSer473/total AKT; ordinary 1-way ANOVA are presented. **(C)** Quantitative analyses of the optical densities for Claudin2 Western Blot bands normalized to β-actin are shown; ordinary 1-way ANOVA. **(D)** Western Blot of Caco2^WT^ for p-AKT^Ser473^ and total AKT for the different experimental conditions are shown. Experiment shown is representative for n=4. **(E)** Quantitative analyses of the optical densities for p-AKT^Ser473^/total AKT; ordinary 1-way ANOVA. Asterisks mark significant difference *p < 0.01, **p < 0.001, ***p < 0.0001, ****p < 0.00001). Comparisons are indicated by the lines above the columns.

Previously it was reported that inflammation-induced Claudin2 upregulation in intestinal epithelial cells is dependent on PI-3-kinase/AKT signaling (29–31). Based on this, we first verified that inhibition of PI-3-kinase using LY294002 blocked TNFα-induced upregulation of Claudin2 (**Figure 6**). In line with previous data, LY294002 attenuated the TNFα-induced phosphorylation of AKT at Ser 473 in Caco2 Dsg2^WT^ and upregulation of Claudin2 (**Figure 6**). In Caco2 Dsg2^-/-^ where basal Claudin2 expression was increased, augmented basal phosphorylation of AKT^Ser473^ was observed (**Figures 6A–C**). Application of TNFα strongly increased phosphorylation of AKT^Ser473^ which was diminished after application by LY294002 (**Figures 6A, B**). Phosphorylation of AKT^Thr308^ was unaffected under all experimental conditions (**Figure 6A**).

Taken together these data confirmed a critical role of PI-3/AKT signaling in the regulation of Claudin2. Furthermore the increased basal phosphorylation of AKT^Ser473^ followed by an exaggerated phosphorylation pattern after TNFα incubation in Dsg2-deficient cells pointed to a potential role for Dsg2 in modulating PI-3-kinase. To substantiate this conclusion we tested whether application of TP would affect phosphorylation of AKT^Ser473^. As outlined above incubation of Caco2 monolayers resulted in augmented phosphorylation of AKT^Ser473^ to 1.6 ± 0.1-fold of controls. This effect was blocked when Caco2 cells were incubated with TNFα together with TP (1.1 ± 0.1-fold of controls), (**Figures 6D, E**).

### Dsg2 Sequesters PI-3-Kinase Under Basal Conditions

**Figure 7:**
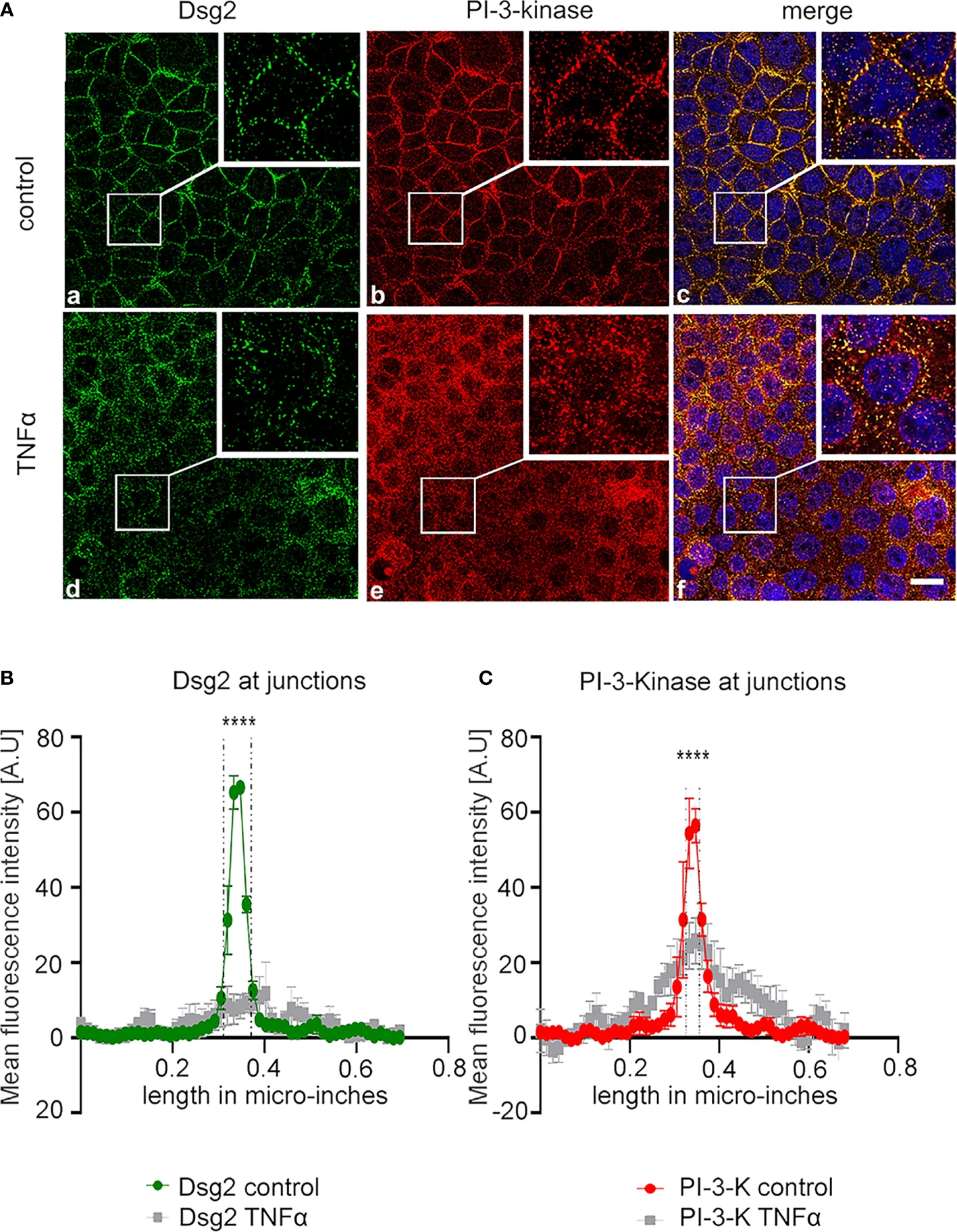
Dsg2 sequesters PI3-kinase under basal conditions. **(A)** Immunostaining for Dsg2 (a,d)=and PI-3-kinase Caco2 cells under control conditions (a-c) and following treatment with TNFα for 24h at 100 ng/ml (d-f) are shown. Merge images include staining with DAPI to visualize cell nuclei (c,f). Images shown are representative for n=4 experiments, scale bar is 2 βµm. **(B, C)** Quantification of all experiments for the conditions of the immunostaining images shown in A are presented; dashed lines in the graphs indicate the area of cell junctions; Asteriks mark significant difference ****p < 0.00001; two way ANOVA.

To further resolve the potential contribution of Dsg2 to regulate both Claudin2 expression and PI-3-kinase signaling we next performed co-immunostaining of Dsg2 and PI-3-kinase under basal conditions and following stimulation with TNFα in Caco2^WT^ cells (**Figure 7A**). Under basal conditions both Dsg2 and PI-3-kinase p110ß were found at the cell borders where they co-localized (**Figure 7Aa–c**). Incubation with TNFα resulted in loss of Dsg2 at the cell borders and redistribution of PI-3-kinase into the cytoplasm (**Figure 7Ad–f**). Accordingly, the co-localization of Dsg2 and PI-3-kinase was abrogated following application of TNFα (**Figure 7Af**). This visual impression was confirmed when quantifications of the immunostaining were carried out. Under basal conditions the mean peak of the staining intensity was found at the cell borders for both Dsg2 and PI-3-kinase. Application of TNFα resulted in a flattening of the curves for Dsg2 and PI-kinase indicating a redistribution of both proteins from the cell borders in the cytoplasm (**Figures 7B, C**).

**Figure 8:**
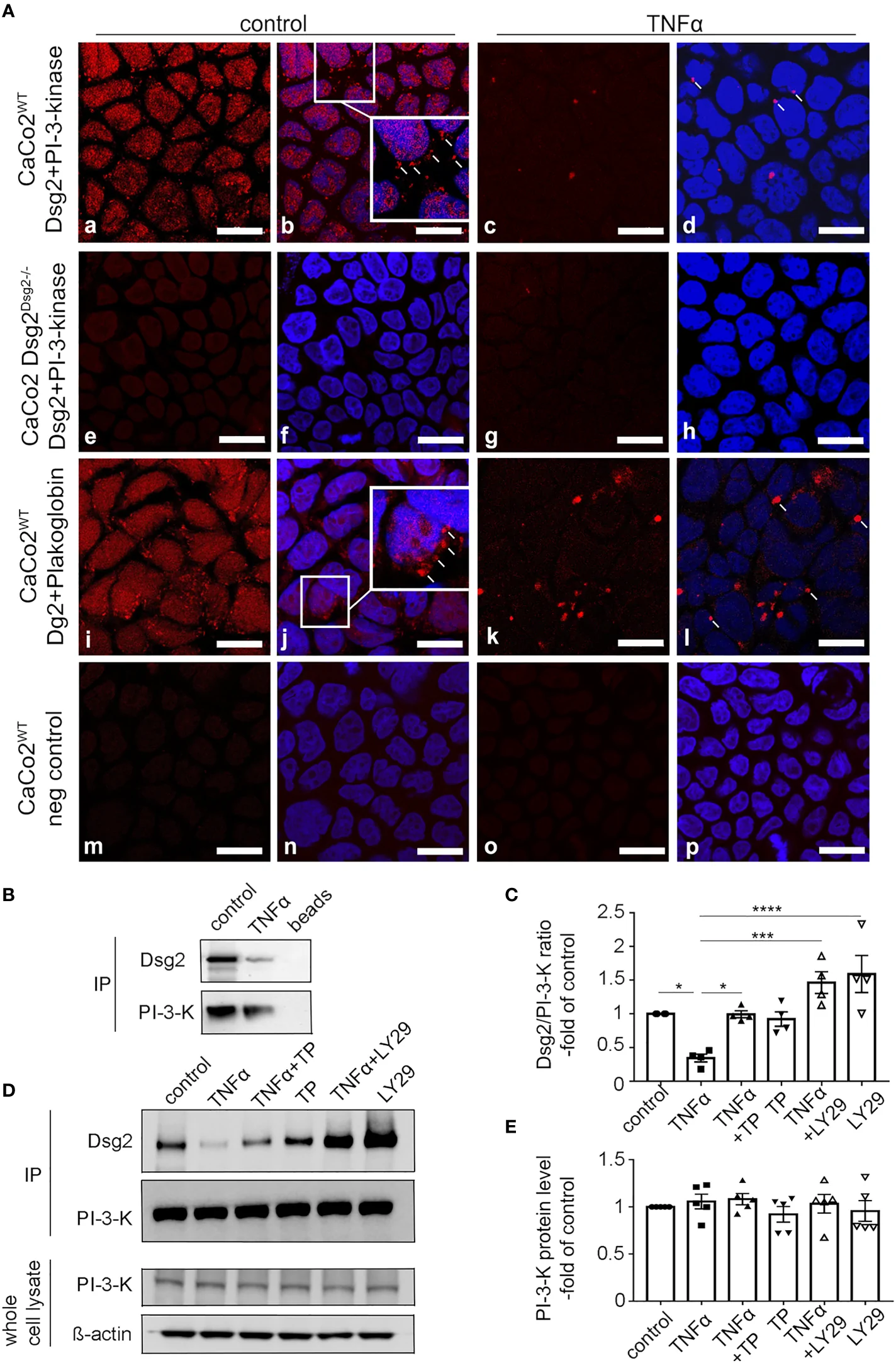
Dsg2 and PI-3-K show direct interaction. **(A)** Images following Proximity ligation assays (PLA) are shown. Images were stained with DAPI to visualize cell nuclei (b, f, j, n, d, h, l). In Caco^WT^ (a-d) red fluorescent spots within cells are shown after incubation of cells with antibodies directed against Dsg2 and PI-3-kinase under basal conditions (a, b). Insert in (b) highlights spots at the cell periphery. Incubation of monolayers with TNFα reduced the number of spots (c, d). PLAs using Caco^Dsg2-/-^ (e-h) under the same conditions and Caco^WT^ without primary antibodies (m-p) served as negative controls. Caco^WT^ were stained with Dsg2 and Plakoglobin as positive control (i-l); images shown are representative for n=5 experiments. Scale bar is10µm. **(B)**, **(C)** Co-Immunoprecipitations (IP) are shown that were performed with 2.5µg PI-3-kinase antibody in Caco2^WT^ cells under control conditions, incubated for 24h with TNFα (100 ng/ml), TP (20 µM), LY294002 (20 µM, PI-3-kinase inhibitor) or in combination. Beads without primary antibody served as negative controls. Western Blot of PI-3-kinase from whole cell lysates of Caco2 Dsg2^WT^ is also shown. ß-actin served as loading control. **(D)** Quantifications of all IPs from **(C)** are presented. Quantifications were carried out by calculating Dsg2/PI-3-kinase ratio from the ODs; n= 4 experiments, Ordinary 1-way ANOVA Asteriks mark significant differences *p < 0.01, ***p < 0.0001, ****p < 0.00001; comparisons are indicated by the lines above the columns. **(E)** Quantification of PI-3-kinase protein level showed no significant difference, n=5, Ordinary 1-way ANOVA.

Since this suggested a direct interaction of Dsg2 and PI-3-kinase we performed proximity ligation assays and co-immunoprecipitation studies. Proximity ligation assays revealed a direct interaction of Dsg2 and PI-3-kinase under basal conditions as revealed by the fluorescent spots throughout the cytoplasm (**Figure 8Aa**) and at the cell periphery (**Figure 8Ab**; inset). This interaction was reduced following incubation of Caco2^WT^ cells with TNFα (**Figure 8Ac, d**). To ensure the staining specificity, the same experiments were carried out using Caco2 Dsg2^-/-^ cells, where no spots i.e. no interaction between Dsg2 and PI-3-kinase was detectable both under basal conditions (**Figure 8Ae, f**) and after incubation with TNFα (**Figure 8Ag,h**). PLAs were also performed in Caco2^WT^ with Dsg2 and the desmosomal plaque protein plakoglobin which is a known interaction partner of Dsg2 (32) (**Figure 8i–l**). This confirmed an interaction between Dsg2 and Plakogblobin (**Figure 8i**, inset in j) under basal conditions which was reduced following incubation of Caco2 cells with TNFα. Negative controls to exclude unspecific staining patterns induced by the duolink fluorescent detection reagent (**Figure 8Am–p**) were performed without application of primary antibodies. This did not result in the visualization of any spots.

Co-immunoprecipitation (IP) using cell lysates from Caco2 Dsg2^WT^ confirmed the interaction of Dsg2 and PI-3-kinase under basal conditions (**Figures 8B–D**) whereas application of TNFα led to decreased interaction of these two proteins. In quantifications of the IPs TNFα reduced the interaction between TNFα and PI-3-kinase to 0.34 ± 0.06-fold of controls whereas incubation of TP with TNFα restored the interaction to control levels (0.99 ± 0.05-fold of controls) and TP alone had no effect (0.92 ± 0.11-fold of control). LY294002 blocked even augmented the interaction between Dsg2 and PI-3-kinase when applied alone (1.6 ± 0.27-fold of control) and when applied together with TNFα (1.5 ± 0.16-fold of control) (**Figure 8D**). Quantification of PI-3-kinase protein levels in whole cell lysates of Caco2 Dsg^WT^ showed no significant differences under the different experimental conditions (**Figures 8C, E**).

## Discussion

The present study extends our previous investigations on the role of desmosomal adhesion and signaling in the regulation of IEB integrity. Here, we show that Dsg2 at the cell borders is required to inhibit upregulation of pore-forming tight junction protein Claudin2. Loss of Dsg2-mediated adhesion induced by antibodies, TNFα or knock-out of Dsg2 induces the activation of PI-3-kinase/AKT signaling which results in the upregulation of Claudin2. This can be explained by the observation that Dsg2 sequesters PI-3-kinase under basal conditions keeping PI-3-kinase/AKT signaling axis in an inactive state. This shows a new mechanism how desmosomal integrity is involved in the regulation of tight junctions in intestinal epithelium.

### Stabilization of Dsg2 by TP Reduced Intestinal Permeability in Acute DSS Colitis *In Vivo*

The stability of intestinal barrier function has been increasingly reported to be dependent on desmosomal integrity in particular by Dsg2-mediated adhesion and signaling (6, 7, 10, 13). Based on this, it has also been proposed that strengthening Dsg2-mediated adhesion between enterocytes might be an attractive target for intestinal barrier stabilization under conditions of inflammation (3). This notion is now substantiated by our present data using the acute DSS-colitis model in which we clearly demonstrate a barrier-protective effect following application of TP *in vivo.* This was accompanied by an improved disease activity score *in vivo* and by reduced overall inflammation as revealed by H.E.-staining. The observation that TP effectively strengthens IEB function and thereby reduces intestinal inflammation supports the overall clinical importance of intestinal barrier integrity and the impact of Dsg2-mediated adhesion as an important player in this context (2, 3, 33).

TP was originally designed as 2 cyclized peptides connected through a flexible amino hexan linker to bind to Dsg1 and was later confirmed to bind Dsg2 as well because of its high sequence homology (10, 34). Since it has been shown that E-cadherin binds Dsg2 on the E-cadherin cis binding interface which appears to be important for desmosome assembly (35) it is possible that TP also binds E-cadherin and thereby strengthens cellular adhesion. On the other hand previous data demonstrated that TP did not modulate homophilic binding of classical cadherins such as E‐cadherin or N‐cadherin (34, 36). The fact that loss of Dsg2 was attenuated using TP both in immunostaining and in Western blot analyses suggests that crosslinking of neighboring Dsg2 proteins may already be sufficient to prevent loss of intercellular adhesion and the induction of barrier-compromising signaling events. This is supported by our *in vitro* data in Caco2 cells, where TP attenuated loss of intercellular adhesion in dispase-based enterocyte dissociation assays and by the fact that loss of intercellular adhesion induced by the application of antibodies directed against the extracellular domain of Dsg2 was sufficient to increase permeability of Caco2 monolayers. Comparable observations were made in previous studies (6, 11).

### Loss of Dsg2 and of Dsg-2-Mediated Adhesion Are Directly Linked With Claudin2 Expression

An interplay between desmosomes and tight junction integrity has been discussed in the recent years although detailed mechanistic insights remained absent (37). It has been proposed that the control of microtubule stabilization by desmosomes could promote the trafficking of tight junction proteins (38). In the intestine, it can be assumed that a mechanical break of tight junctions as a consequence of reduced Dsg2-mediated adhesion may induce increased intestinal permeability. Given the close correlation between loss of tight junctions in general and Dsg2 at the cell borders this appears to be reasonable (6–8, 10). However, the fact that the Claudin2 is upregulated on both, RNA- and protein levels following loss of Dsg2 indicates a mechanistic interplay between these two proteins instead of only a passive interplay of tight junctions. Following this, Dsg2 may serve as a scaffold protein to modulate downstream signaling either in response to other upstream signaling events and/or to changes in cell adhesion. The observation that loss of Dsg2 induced by TNFα, by gene knock out and by antibody-induced loss of intercellular adhesion resulted in increased Claudin2 levels demonstrates that all of these events are sufficient to induce Dsg2-dependent signaling. The role of Dsg2 as a protein critically involved in cellular signaling events has been recognized earlier in the context of apoptosis, where a fragment of the intracellular domain of Dsg2 contributed to the induction of cell death under certain experimental conditions (12, 13, 15). In addition is has been observed that Dsg2 or desmosomes in general contribute to the regulation of Rho GTPase activity by the modulation of Rho GEF and regulate the activity of protein kinase C which was observed in the context of plakophilin2 in keratinocytes and in cardiomyocytes (39–44).

### Dsg2-Mediated Sequestering of PI-3-Kinase Regulates Activity of the PI-3-Kinase/AKT Signaling Axis

Increased Claudin2 levels are a typical hallmark in response to inflammatory stimuli in the intestine (45, 46). According to this, Claudin2 is upregulated under conditions of acute inflammation in DSS-induced colitis in mice (47) and in patients with IBD (9, 10, 48). On a functional level Claudin2 as a pore-forming tight junction protein is critically involved in symptoms such as diarrhea in intestinal inflammation (49). On a mechanistic level it has been demonstrated earlier that upregulation of Claudin2 is dependent on PI-3-kinase activation leading to phosphorylation of AKT. Accordingly, inhibition of PI-3-kinase using LY294002 was effective to attenuate inflammation-induced upregulation in previous studies (30, 31, 50, 51). This supports our current observation where LY294002 blocked TNFα-induced upregulation of claudin2 in Caco2 cells. The TNFα-induced activation of PI-3-kinase may lead to Cdx2 expression as described previously for IL-6 (30). The enhanced expression of the transcription factor Cdx2 may then activate the claudin2 promotor resulting in the increased Claudin2 expression (29). The fact that LY294002 completely blocked both AKT phosphorylation and increased Claudin2 expression indicates that the critical event is upstream of AKT i.e. at the level of PI-3-kinase activation.

The novel observation here is that Dsg2 sequesters PI-3-kinase in intestinal epithelial cells. The finding that we observed increased phosphorylation of AKT^Ser473^ in Dsg2-deficient cells when compared to WT cells under basal conditions and highly increased phosphorylation following application of TNFα led to the assumption that the sequestering of PI-3-kinase by Dsg2 may regulate its activity. On the other hand, the fact that TNFα application still results in increased Claudin2 expression and AKT phosphorylation in Dsg2-deficient cells also points to the presence of Dsg2-independent pathways in addition to the proposed mechanism here. The Dsg2-independent pathways may involve direct AKT phosphorylation by TNFα-induced activation of src kinase (52) by miRNAs (53) and also by integrins as has been observed in platelets (54) upstream of PI-3-kinase. Nonetheless, the experiments using TP to stabilize Dsg2-mediated adhesion showed that this was sufficient to block TNFα-induced AKT-phosphorylation and Claudin2 upregulation which overall supports the notion that Dsg2 is critically involved in the regulation of the PI-3-kinase/AKT signaling axis.

Application of TNFα not only resulted in loss of Dsg2 at the cell borders but also remarkably removed PI-3-kinase to the cytoplasm in immunostaining. The direct interaction of PI-3-kinase and Dsg2 was proven by PLA-assays and co-immunoprecipitation. However, when taking into account the staining pattern in PLA-assays it appears that not only desmosomal Dsg2 at the cell borders but also extradesmosomal Dsg2 which can be found apically in enterocytes also appears to be involved in sequestering PI-3-kinase (11). The presence of extradesmosmal Dsg2 may also explain the dotted staining pattern across the whole cells in PLAs. Interestingly, both TP and LY294002 blocked the dissociation of PI-3-kinase and Dsg2. According to the quantifications of the co-immunoprecipitation assays, LY294002 even increased the interaction between Dsg2 and PI-3-kinase above baseline levels both when applied alone and following application of TNFα. This suggests that activation of PI-3-kinase causes disassembly of both proteins leading to increased Claudin2 expression. On the other hand as outlined above the stabilization of Dsg2 by TP blocked the dissociation of Dsg2 and PI-3-kinase and thereby attenuated AKT phosphorylation. Taking all these observations together, it is conceivable that the sequestering of PI-3-kinase to Dsg2 is required to prevent further downstream signaling in response to PI-3-kinase activation. Therefore, loss of the interaction triggers further downstream signaling. In addition, it can be speculated that loss of Dsg2 may further promote the susceptibility PI-3-kinase to be further activated. Based on this, we suggest that Dsg2 recruits PI-3-kinase under basal conditions. Loss of Dsg2 or Dsg2-mediated adhesion promotes the activation of PI-3-kinase and/or downstream signaling leading to AKT phosphorylation which then results in the upregulation of Claudin2.

In summary, our current data not only show a novel and important mechanism by which Dsg2 i.e. desmosomes are directly involved in the regulation of tight junction proteins but also strengthen the potential role of Dsg2 as a promising therapeutic target to stabilize intestinal barrier function in intestinal inflammation.

## Data Availability Statement

The raw data supporting the conclusions of this article will be made available by the authors, without undue reservation.

## Ethics Statement

The animal study was reviewed and approved by Regierung von Unterfranken.

## Author Contributions

NB: Conceptualization, Investigation, Data analysis, Methodology, and Writing original draft. MM: Investigation, Data analysis, and Methodology. FK: Investigation and Data analysis. CO: Investigation and Data analysis. MP: Investigation and Data analysis. C-TG: Supervision, Data analysis, and Resources. JW: Conceptualization, Supervision, Data analysis, and funding acquisition. NS: Conceptualization, Supervision, Data analysis, Funding acquisition, Writing original draft, and review and editing. All authors contributed to the article and approved the submitted version.

## Funding

These studies were funded by Deutsche Forschungsgemeinschaft (DFG) Priority Programm SPP 1782 to NS and JW.

## Correction note

This article has been corrected with minor changes. These changes do not impact the scientific content of the article.

## Conflict of Interest

The authors declare that the research was conducted in the absence of any commercial or financial relationships that could be construed as a potential conflict of interest.

## Publisher’s Note

All claims expressed in this article are solely those of the authors and do not necessarily represent those of their affiliated organizations, or those of the publisher, the editors and the reviewers. Any product that may be evaluated in this article, or claim that may be made by its manufacturer, is not guaranteed or endorsed by the publisher.
