# Correction: Desmoglein2 regulates Claudin2 expression by sequestering PI-3-Kinase in intestinal epithelial cells

**DOI:** 10.3389/fimmu.2026.1992331

**Published:** 2026-10-08

**Authors:** Natalie Burkard, Michael Meir, Felix Kannapin, Christoph Otto, Maximilian Petzke, Christoph-Thomas Germer, Jens Waschke, Nicolas Schlegel

**Affiliations:** 1 Department of General, Visceral, Transplant, Vascular and Pediatric Surgery University Hospital Würzburg, Würzburg, Germany; 2 Institute of Anatomy and Cell Biology, Department I, Ludwig-Maximilians-Universität München, Munich, Germany

**Keywords:** Claudin2, Dsg2, inflammation, intestinal barrier, PI-3-kinase, inflammatory bowel disease, desmosome, tight junction

There was an error in **Figure 2Ac** as published. An in correct representative H&E image was inadvertently included for the DSS+TP group.

The incorrect image has been replaced with the correct representative H&E image from the DSS+TP group. The corrected **Figure 2Ac** appears below. This correction does not affect the results or conclusions of the article.

**Figure 2:**
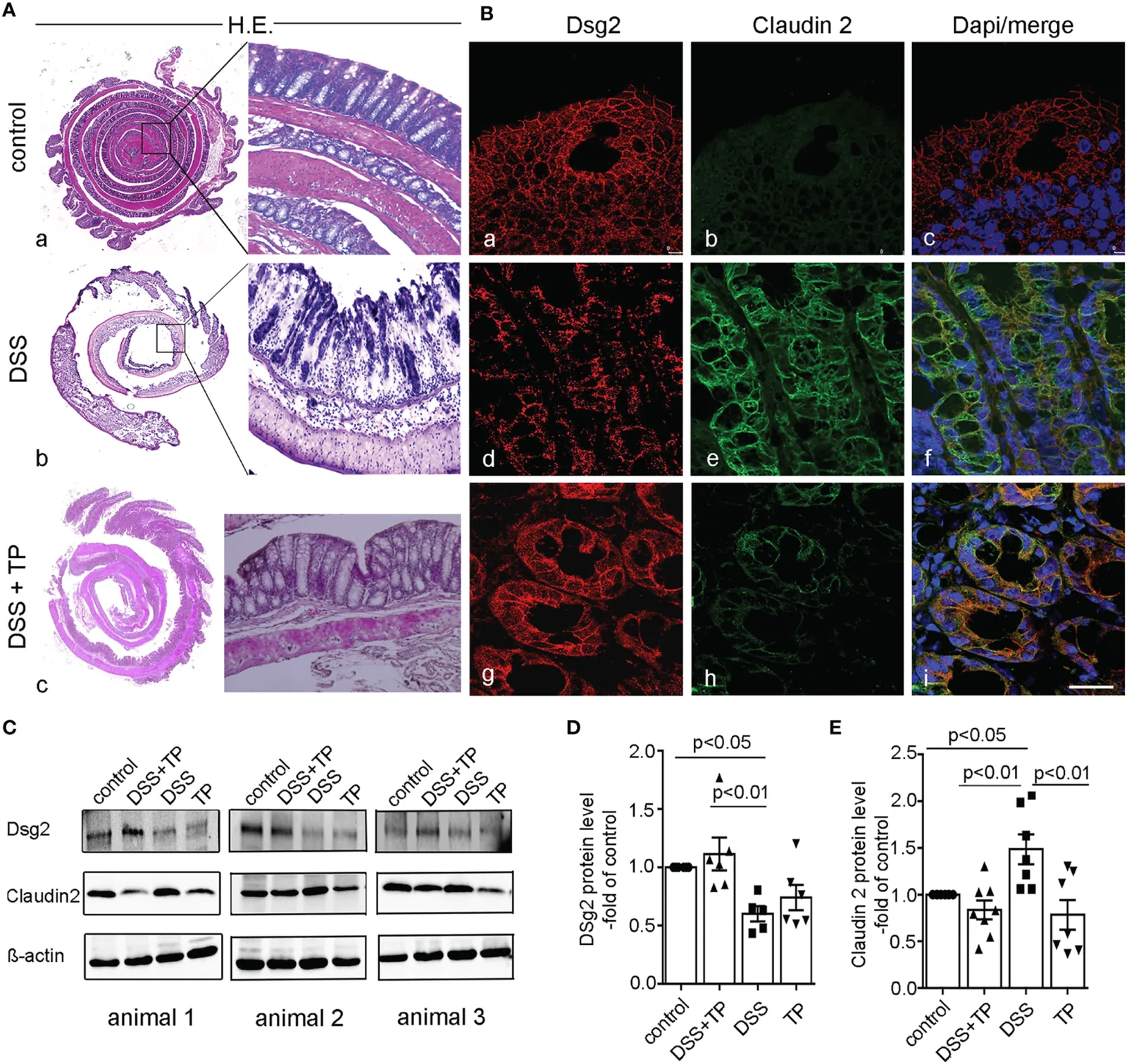
TP affected DSS-induced redistribution of Dsg2 and Cld2 at the cell border in vivo. **(A)** Representative H&E staining of mouse colon are shown (left overview; right magnification). Controls (a) show a normal morphology of the gut whereas DSS (2.5% DSS) animals (b) show signs of severe inflammation with mucosal damage. Treatment of animals with 10 µM TP (c; DSS+TP) attenuated inflammation-induced changes in the colon. Images shown are representatives for n=6 in each group. **(B)** Immunostaining for Dsg2 (a, d, g), Claudin2 (b, e, h) or merged images with staining of cell nuclei using DAPI (c, f, i) are shown for the same conditions. The loss of Dsg2 in DSS animals (d) compared to controls (a) is attenuated by TP treatment (g). This is paralleled by augmented Claudin2 staining (e) in DSS colitis compared to controls (b) whereas TP blocked inflammation-induced increase of Claudin2 (h). Images shown are representatives of n=6 in each group. Scale bar is 25 µm. **(C)** Western Blots from colon lysates of control animals, animals with DSS colitis treated with TP (DSS+TP), DSS colitis alone and with TP alone are shown for Dsg2 and Claudin2 and β-actin. A set of 3 different animals is shown. **(D)** Quantitative analyses of the optical densities for Dsg2 Western blot bands normalized to β-actin showed reduced Dsg2 compared to DSS-colitis. This reduction was not observed in DSS+TP-treated animals (p<0.05 compared to the control group, n=6; p<0.01 compared to DSS+TP-treated mice; TP showed no significant change compared to all other groups; n=6 for each group, ordinary 1-way ANOVA). **(E)** Quantitative analyses of the optical densities for Claudin2 Western Blot bands normalized to β-actin showed significantly increase of Claudin2 in DSS mice compared to control mice (p<0.05) which was blocked by TP (p<0.01) (n=6 for each group; ordinary one-way ANOVA).

The original version of this article has been updated.

